# Editorial: Genetic and environmental roles in bone and joint diseases

**DOI:** 10.3389/fgene.2023.1177191

**Published:** 2023-04-21

**Authors:** Mikko J. Lammi, Xi Wang, Yujie Ning

**Affiliations:** ^1^ Department of Integrative Medical Biology, Umeå University, Umeå, Sweden; ^2^ School of Public Health, Xi’an Jiaotong University Health Science Center, Key Laboratory of Trace Elements and Endemic Diseases, National Health and Family Planning Commission, Xi’an, China

**Keywords:** genetics, environment, bone, joint, cartilage

Various types of multiomic analyses have advanced the biomedical knowledge significantly during the recent years, and we can expect novel findings based on those observations reach clinical practices as well. Still the roles of environmental factors behind the diseases have to be considered as well.

The goal of the call for this Research Topic *Genetic and environmental roles in bone and joint diseases* was to reveal novel components involved in diagnosis and therapy, ethiology and pathogenesis of the diseases in bone and joint diseases. Also reports on responses to environmental exposure by different approaches were welcome. The Research Topic mainly focuses on bone and joint diseases, such as osteoporosis, osteoarthritis, inflammatory arthritis, bone metastasis, osteonecrosis, endemic Kashin-Beck disease, and Marfan syndrome. The call attracted 23 submitted manuscripts, of which 7 were accepted for publication after rounds of revisions. These papers are summarized below.

It has been shown previously that osteoprotegerin gene (*OPG*) has relationship to pathogenesis of osteoporosis, and *OPG* gene polymorphisms have been identified (Li et al., 2021). Still the previous studies have given inconsistent conclusions. A meta-analysis study by Han et al. was performed to gain more accurate and reliable evidence on the association. The conclusion was that associations of the *OPG* A163G and G1181C polymorphisms and osteoporosis risk are false-positive rather than true associations. This is an important amendment to our understanding on the role of *OPG* gene association with osteoporosis.

Another meta-analysis study by authors Liu et al. was also carried out to verify previous inconsistent results on correlation of cartilage intermediate layer protein gene (*CILP*) and IL-1α (IL1A) polymorphisms with the susceptibility of intervertebral disc disease (IDD). The investigated *CILP* polymorphism was previously shown to be associated with the IDD susceptibility in Japanese population (Seki et al., 2005), while in Finnish or Chinese cohorts no such association was observed (Virtanen et al., 2007). This study showed *CILP* polymorphism association with radiologic intervertebral degeneration, while association between IL-1α polymorphism and radiologic or symptomatic intervertebral disc degeneration was less obvious, complementing the previous conclusions.

Nutritional components, especially those used daily, expose us to chemicals, which may have either healthy or harmful effects. Tea is a commonly used stimulant containing several chemicals, which are potential effectors of osteoarthritis (OA). Indeed, a 5-year cohort study suggested that higher consumption of green tea was associated with lower incidence of knee OA in males but not in females (Takiguchi et al., 2019), although this is not a consistent finding. The authors Li et al. in their article were interested in studying the relationship between tea consumption and OA. Analyzing some common single-nucleotide polymorphisms they made the finding that higher daily consumption of tea increases the risk of OA, which is inconsistent with previous results. Thus, the results warrant further studies to provide important knowledge refinements on the properties of tea supplement.

Microgravity during the space flights is known to lead to bone loss particularly in the load-bearing bones. The authors Wang et al. used bioinformatics of three transcriptome datasets to reveal molecular mechanisms related to microgravity-induced bone loss. They could identify 11 hub genes and some miRNA-mRNA interactions, which can give rise to new ideas on mechanoregulation and tissue engineering approaches. This is an excellent example exploiting the pre-existing gene expression data to gain deeper understanding otherwise but possible or producing high cost to gather the data.

Kashin-Beck disease is a degenerative osteoarthropathy, found mainly in China today. Its symptoms show similarities with OA, but it has a remarkably different etiology and pathogenesis (Guo et al., 2014). Its pathogenesis is not known in detail, thus, the research on this disease has been active the last 10–15 years. In article by Yang et al., the authors studied the histopathological changes, transciptomics and differentially expressed miRNAs of subchondral bone between Kashin-Beck patients and those with OA. Several novel genes were observed to have differential expressions, and they were considered to have potential as differential diagnostic biomarkers between Kashin-Beck disease and OA. This is important since we would need better biomarkers to early detection of KBD. This study indicates that the articular joint disease is a complicated entity, with disease symptoms also specific to tissues other than articular cartilage.

Marfan syndrome was in focus of two other articles. The authors Chen et al. review the recent advances in the genotype-phenotype correlations of Marfan syndrome and related fibrillinopathies. It presents topical knowledge on pathogenic variants in fibrillin 1 (*FBN1*) gene, and lists the potential molecular bases and mechanisms related to Marfan syndrome, supplementing genotype-phenotype correlations lacking so far.

Despite the known pathogenic variants of Marfan syndrome, the genetic cause in about 10% of the patients with typical Marfan phenotypes cannot be identified at present (Ziegler et al., 2021). The authors Hu et al. performed a functional analysis of an intronic variant of fibrillin1 gene (*FBN1*). DNA analysis of a 32-year-old patient with typical Marfan syndrome symptoms revealed an intronic variant of the FBN1 gene, which was predicted to affect RNA splicing. The variant also abolished the canonical splicing site of intron 3, which leads to activation of two cryptic splicing sites. This study further improves our knowledge of the genetic spectrum of Marfan syndrome and fibrillinopathies.

## References

[B1] Guo X. Ma W. J. Zhang F. Ren F. L. Qu C. J. Lammi M. J. (2014). Recent advances in the research of an endemic osteochondropathy in China: Kashin-Beck disease. Osteoarthr. Cartil. 22 (11), 1774–1783. 10.1016/j.joca.2014.07.023 25106677

[B2] Li X. Cheng J. Dong B. Zhao X. Zhou Z. (2021). Common variants of the *OPG* gene are associated with osteoporosis risk: A meta-analysis. Genet. Test. Mol. Biomarkers 25 (9), 600–610. 10.1089/gtmb.2020.0282 34515523

[B3] Seki S. Kawaguchi Y. Chiba K. Mikami Y. Kizawa H. Oya T. (2005). A functional SNP in CILP, encoding cartilage intermediate layer protein, is associated with susceptibility to lumbar disc disease. Nat. Genet. 37 (6), 607–612. 10.1038/ng1557 15864306

[B4] Takiguchi R. Komatsu R. Kitamaru K. Watanabe Y. Kobayashi O. R. Kobayashi R. (2019). Modifiable factors associated with symptomatic knee osteoarthritis: The murakami cohort study. Maturitas 128, 53–59. 10.1016/j.maturitas.2019.06.013 31561824

[B5] Virtanen I. M. Song Y. Q. Cheung K. M. C. Ala-Kokko L. Karppinen J. Ho D. W. H. (2007). Phenotypic and population differences in the association between CILP and lumbar disc disease. J. Med. Genet. 44 (4), 285–288. 10.1136/jmg.2006.047076 17220213 PMC2598035

[B6] Ziegler S. M. Sloan B. Jones J. A. (2021). Pathophysiology and pathogenesis of Marfan syndrome. Adv. Exp. Med. Biol. 1348, 185–206. 34807420 10.1007/978-3-030-80614-9_8 PMC8915437

